# Chlorpromazine Efficiently Treats the Crisis of Pheochromocytoma: Four Case Reports and Literature Review

**DOI:** 10.3389/fcvm.2021.762371

**Published:** 2021-11-22

**Authors:** James Jiqi Wang, Zuowen He, Yan Yang, Bo Yu, Hong Wang, Hu Ding, Guanglin Cui, Luyun Wang, Dao Wen Wang, Jiangang Jiang

**Affiliations:** ^1^Division of Cardiology, Department of Internal Medicine, Tongji Medical College, Tongji Hospital, Huazhong University of Science and Technology, Wuhan, China; ^2^Hubei Key Laboratory of Genetics and Molecular Mechanism of Cardiological Disorders, Wuhan, China; ^3^Division of Endocrinology, Department of Internal Medicine, Tongji Medical College, Tongji Hospital, Huazhong University of Science and Technology, Wuhan, China

**Keywords:** pheochromocytoma multisystem crisis, chlorpromazine, pheochromocytoma, case series, hypertension, catecholamine

## Abstract

Pheochromocytoma multisystem crisis (PMC) is a potentially lethal emergency due to catecholamine secretion. The condition manifests as severe hypertension to intractable cardiogenic shock and has a high mortality rate. This study explored the efficacy and safety of applying chlorpromazine on PMC patients. The study included seven patients (median age, 42 years; range, 14–57 years) diagnosed with pheochromocytoma. Four consecutive PMC patients were admitted to our critical care unit between 2016 and 2020 due to abdominal or waist pain, nausea, and vomiting. Their blood pressure (BP) fluctuated between 200–330/120–200 and 40–70/30–50 mmHg. Chlorpromazine (25 or 50 mg) was injected intramuscularly, followed by continuous intravenous infusion (2–8 mg/h). The patients' BP decreased to 100–150/60–100 mmHg within 1–3 h and stabilized within 3–5 days. Two weeks later, surgical tumor resection was successfully performed in all four patients. Similar clinical outcomes were also obtained in three patients with sporadic PMC reported in the literature who received chlorpromazine treatment, which reduced their BP readings from >200/100 mmHg to 120/70 mmHg. Our observations, combined with sporadic reports, showed that chlorpromazine efficiently controlled PMC. Thus, future studies on the use of chlorpromazine are warranted.

## Introduction

Pheochromocytomas (PHEOs) and extra-adrenal paragangliomas (EAPs) are rare chromaffin cell tumors originating from adrenal chromaffin cells or extra-adrenal chromaffin cells in sympathetic and parasympathetic paraganglia, respectively (1). They are associated with catecholamine secretion and are assessed optimally by measurements of plasma or urinary metanephrine and normetanephrine levels (2, 3). PHEOs are rare, with an estimated incidence of 0.005–0.1% in the general population (4, 5); however, the mortality is as high as 26% (6).

The symptoms of PHEO can vary greatly and most are due to the direct action of catecholamine secretion (3). Predominant clinical presentations include hypertension (80.7%), palpitations (59.3%), headache (60.4%), and diaphoresis (52.4%) (7). In some cases, catecholamine overproduction in short time can result in pheochromocytoma multisystem crisis (PMC), characterized by severe hypertension, circulatory failure, and shock, which involve with subsequent involvement of multiple organ systems, including the cardiovascular, pulmonary, neurological, gastrointestinal, renal, hepatic, and metabolic systems as described by Scholten et al. and Tschuor et al. (8, 9). During PMC, the patient's temperature can exceed 40°C and the patient presents with hypertension with frequently alternating episodes of hyper- and hypotensive cyclic periods, multiple organ damage, and encephalopathy (10). Prolonged exposure to excess levels of epinephrine and norepinephrine can result in hypovolemia caused by vasoconstriction of the artery and vein (11). Increased blood pressure (BP) stimulates baroreceptors and triggers negative feedback, decreasing peripheral vascular resistance and cardiac output (12). Both hypovolemia and the negative feedback of baroreceptors contribute to the rapid variation between hypertension and hypotension (12). Delayed or improper treatment of PMC can lead to severe adverse sequelae (13). Surgical excision is the most efficient treatment for PHEOs (1). Moreover, optimal preoperative medication to stabilize patient hemodynamics is essential; otherwise, PHEO resection is associated with increased incidence of hypertensive crisis and death (14).

Alpha-adrenoceptor blockers are recommended for PMC control; additionally, calcium channel blockers and sodium nitroprusside are also frequently used before surgical treatment for patients with PHEOs (2). However, in clinical practice, some patients respond poorly to these rescue measures and sodium nitroprusside may cause irreversible hypotension. Therefore, an ideal therapeutic regimen or drug is required for the management of patients with PMC. Among potential treatments, chlorpromazine provides sedation, antipsychotics, and other effects, unlike other α-blockers (15). Therefore, we investigated whether chlorpromazine could be safely and effectively applied for the treatment of PMC. This study presented four PMC patients (Table 1) meeting the above criteria and reviewed three PMC cases successfully treated with chlorpromazine in literature (16, 17). Our results support future studies on the use of chlorpromazine and provide an alternative medication that is potentially safe and effective for the treatment of PMC.

**Table 1:** Patients' blood pressure and organ function function during chlorpromazine treatment.

	**Patient 1**		**Patient 2**		**Patient 3**		**Patient 4**	
**BP variation**								
BP (mmHg) at baseline	100/60–330/150		80/50–270/130		80/40–179/111		60/40–240/120	
BP (mmHg) 3 hrs after CPZ	107/70–280/150		106/72–208/110		109/60–133/80		94/66–179/120	
BP (mmHg) 3–5 days after CPZ	100/60–125/70		115/50–130/70		100/50–120/70		100/60–120/80	
BP (mmHg) after tumor extirpation	98/61–126/80		100/55–116/60		100/50–110/60		110/60–120/70	
**Organ function and SpO2**	**Before CPZ**	**After CPZ**	**Before CPZ**	**After CPZ**	**Before CPZ**	**After CPZ**	**Before CPZ**	**After CPZ**
SpO2 (%)	96	99–100	94	99–100	96	99–100	93	99–100
CNS symptom	Severe headache Unconscious	Normal	Severe headache	Normal	Severe headache	Normal	Severe headache	Normal
NT-proBNP (pg/mL)	778	–	34,082	299	11,596	5,307	31,018	738
Hs-cTNI (pg/mL)	371.3	–	153.1	9.8	27,429.5	354.9	212	16.2
EF (%)	–	–	58	–	45	65	56	60
AST (U/L)	166	79	38	23	65	31	163	113
ALT (U/L)	78	37	34	14	47	32	67	31
ALB (g/L)	50	37.7	48.7	38.7	41.6	30.3	30.5	34.4
Alkaline Phosphatase (U/L)	170	80	96	60	54	42	175	61
Y-Glutamyl Transpeptidase (U/L)	123	70	27	18	32	27	84	120
Cr (umol/L)	122	73	77	36	138	55	73	56
BUN (mmol/L)	8.46	8.9	14.06	3.96	7.38	5.12	11.3	2.4
HCO3- (mmol/L)	25.3	24.9	18.6	26	18.6	21.5	28.2	25.6
eGFR (ml/min/1.73 m∧2)	62.6	108.8	74.1	115.3	41.2	113	79.0	99.7
Urine protein	±	–	+++	–	+	±	±	–
Urine erythrocyte	+	±	+++	++	+++	++	++	–

*CPZ, chlorpromazine' BP, blood pressure; SpO_2_, oxygen saturation; CNS, central nerve system; NT-proBNP, amino-terminal pro-brain natriuretic peptide; Hs-cTNI, High-sensitivity troponin I (pg/mL); EF, ejection fraction; AST, aspartate aminotransferase; ALT, alanine aminotransferase; ALB, albumin; Cr, creatinine; BUN, blood urea nitrogen; eGFR: estimated glomerular filtration rate*.

## Case Reports

**Figure 1:**
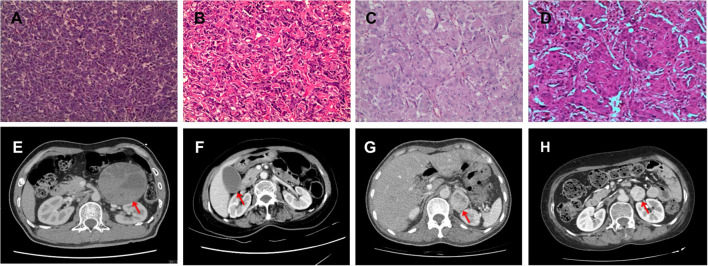
Pathology and CT images of the tumor **(A,E)**: Patient 1; **(B,F)**: Patient 2; **(C,G)**: Patient 3; **(D,H)**: Patient 4. Red arrow indicate tumor mass.

**Patient 1**. A 42-year-old man presented with subcostal pain persisting for 1 week. Each episode lasted for 2–3 min and eventually resolved. He had a history of gastric ulcers and hypertension. Hypertension was poorly controlled (up to 160/95 mmHg) with oral irbesartan. Electronic gastroscopy revealed superficial gastritis. However, symptoms were not relieved after treatment for gastritis. On admission, his body temperature was 36.5°C, BP was 104/74 mmHg, pulse rate was 100 bpm, and respiratory rate was 20/min. Catecholamine metabolite levels were also elevated (urinary norepinephrine: 1927.2 μg/24 h; epinephrine: 73.0 μg/24 h; urinary VMA: 453.6 umol/24 h). Computed tomography (CT) showed a 8.9 cm x 8.1 cm round left adrenal mass (Figure 1E).

**Figure 2:**
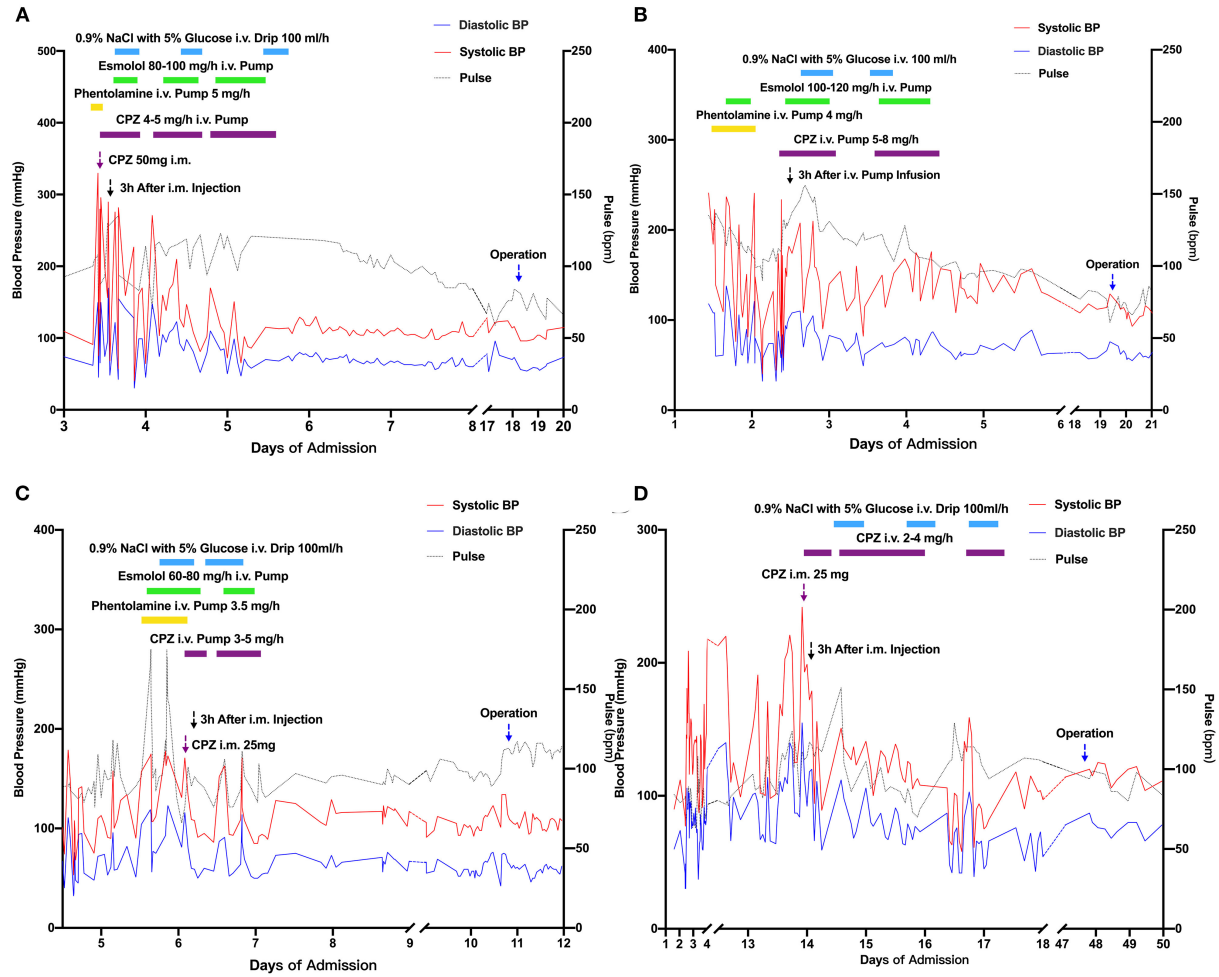
Curves of systolic and diastolic blood pressure and heart rate during chlorpromazine application **(A)**: Patient 1; **(B)**: Patient 2; **(C)**: Patient 3; **(D)**: Patient 4. Colored bars represent continuous intravascular infusion (i.v. pump). Purple arrows indicate time of bolus intramuscular injection of chlropromazine. Black arrows indicate 3 h after chlorpromazine application. Blue arrows indicate time of operation. BP, blood pressure; CPZ, chlorpromazine; i.v., intravenous; i.m., intramuscular.

During hospitalization, the patient's BP fluctuated remarkably (Figure 2A). On the third day, his BP reached 330/150 mmHg, pulse rate was 108 bpm, respiratory rate was 22/min and body temperature reached 40°C (Table 1), along with salivation and diaphoresis. The patient suffered from severe headache and lost consciousness for ~1 min. Based on his fierce BP fluctuation and mental stasis combined with clearly elevated urine catecholamine metabolite and CT findings, the patient was diagnosed with PMC, which was in accordance with the previously mentioned symptoms (Table 1). He was immediately administered phentolamine mesylate to control his BP; however, his response was poor. Intramuscular injection (50 mg) followed by continuous infusion of chlorpromazine (4–5 mg/h) was administered, after which BP gradually dropped to 102/70 mmHg (Figure 2A). Esmolol and volume expansion with glucose saline were also simultaneously administered. The frequency of hypertensive episodes and BP amplitude decreased gradually. After 2–3 days, the patient's BP and heart rate stabilized and no further PMC episodes occurred (Figure 2A). On the 18th day, he underwent left adrenalectomy, during which a 9 cm × 7 cm × 4 cm adrenal mass was removed. Pathological examination confirmed the diagnosis of PHEO (Figure 1A). Postoperatively, the patient's BP was 98–126/61–80 mmHg and his heart rate was 61–88 bpm (mostly around 70 bpm). Plasma normetanephrine and blood metanephrine levels returned to normal ranges (normetanephrine: 0.08 nmol/L; metanephrine: <0.07 nmol/L, Supplementary Table 1) and he was discharged on the 27th day of hospitalization.

**Patient 2**. A 57-year-old woman presented with a right adrenal tumor and hypertension (systolic pressure occasionally reached 250 mmHg) 8 years previously without proper treatment, accompanied by burning pain in chest, with palpitations and pulsatile headaches in the supine position that had lasted for 1 year. She began vomiting without apparent cause 2 days before her presentation. The patient was admitted to the emergency department where her BP increased from 121/79 to 222/117 mmHg within 25 min. On admission, her body temperature was 36.5°C, BP was 258/121 mmHg, pulse rate was 132 bpm, and respiratory rate was 20/min. Catecholamine metabolite tests revealed elevated levels of urinary vanillylmandelic acid (VMA) (409.2 μmol/24 h, Supplementary Table 1). CT showed a 4.9 cm x 2.9 cm right adrenal mass (Figure 1F). A diagnosis of PHEOs was made according to patient's fluctuating BP, significantly elevated urinary VMA and CT results (2, 18).

On the second day, she experienced a severe headache, diaphoresis and emesis (Table 1). Body temperature reached 39.5°C and BP fluctuated between 270/130 and 80/50 mmHg (Figure 2B). We immediately realized that a PMC episode had occurred. Phentolamine and esmolol were administered, with a poor response. The patient was then treated with continuous infusion of chlorpromazine (5–8 mg/h). Glucose saline were also administered for volume expansion. Her BP gradually stabilized between 115/50 and 130/70 mmHg, with no subsequent PMC episodes (Figure 2B). Right adrenalectomy was successfully performed on the 19th day of hospitalization. Pathological examination confirmed the diagnosis of PHEO (Figure 1B). After the operation, the patient's BP remained between 100–116 and 55–60 mmHg (Figure 2B), heart rate was 69–80 bpm, and urinary VMA level returned to within the normal range (25.2 μmol/L, Supplementary Table 1). The patient was discharged 6 days after the surgery.

**Patient 3**. A 40-year-old woman experienced chest discomfort, nausea, and vomiting for 2 days after she caught a cold. On admission, her body temperature was 36.0°C, pulse was 150 bpm, and respiratory rate was 14/min. BP was too low to be measured. With a pertinent history of viral myocarditis, we first considered fulminant myocarditis. Her vital signs were extremely unstable. Thus, we administered antiviral, immune supportive, and life-supportive measures in accordance with the procedures previously mentioned (19). The patient's condition gradually improved during therapy. However, during hospitalization, we noticed that her BP fluctuated between 86–168 and 64–117 mmHg. The pulse rate was over 110 bpm. Laboratory tests revealed elevated catecholamine metabolite levels (urinary norepinephrine: 300.3 μg/24 h; epinephrine: 793.0 μg/24 h; VMA: 110.2 μmol/24 h, Supplementary Table 1). CT showed a 2.7 cm × 3.6 cm left adrenal mass (Figure 1G). Combining the patient's laboratory test, result CT of scan and abnormal BP variation, the patient was diagnosed with PHEOs (2, 18).

On the 6th day of admission, the patient experienced severe headaches and emesis (Table 1). BP rapidly reached 220/108 mmHg and automatically dropped to 118/78 mmHg within 5 min (Figure 2C). Pulse was over 100 bmp. Considering that she was previously diagnosed with PHEOs, we concluded that she was suffering from a PMC episode. The patient showed poor response to phentolamine and esmolol, so chlorpromazine (25 mg i.m. and continuous i.v. infusion 3–5 mg/h) and glucose saline were administered as described above. Her symptoms were relieved after 1 h and BP gradually stabilized to around 120–100/70–50 mmHg within the next 2 days (Figure 2C). Surgery was performed on the 10th day. Pathological examination confirmed that the excised tissue was PHEO (Figure 1C). The patient's postoperative BP remained stable at 100–110/50–60 mmHg and catecholamine metabolite levels returned to normal (urinary VMA: 14.8 μmol/24 h; urinary norepinephrine: 15.3 μg/24 h; urinary epinephrine: 2.3 μg/24 h; plasma normetanephrine: 0.13 nmol/L; plasma metanephrine: 0.11 nmol/L, Supplementary Table 1). The patient was discharged on the 18th day.

**Patient 4**. A 57-year-old woman was admitted to the hospital with a complaint of acute vision loss in both eyes occurring within the previous 10 days. She was primarily diagnosed with ophthalmoneuromyelitis and was admitted to the neurology department for further treatment. On admission, her temperature was 36.5°C, pulse was 76 bpm, respiratory rate was 20/min, and BP was 148/102 mmHg. She was administered with neurotrophic factors and methylprednisolone. On the 2nd day of hospitalization, the patient's BP started to fluctuate between 210/110 and 90/60 mmHg (Figure 2D). Further enhanced abdominal CT imaging revealed a high-density tumor measuring 2.6 cm × 2.8 cm in the left adrenal region (Figure 1H). Laboratory tests revealed an increased catecholamine metabolite level (urinary VMA, 65.2 μmol/24 h; urinary epinephrine, 334.0 μg/24 h; urinary norepinephrine: 212.8 μg/24 h, Supplementary Table 1). Based on patient's abnormal vital signs (BP variation, fever and tachycardia), elevated urine catecholamine metabolite and CT test result, she was diagnosed with PHEOs (2, 18).

The patient was transferred to the cardiovascular department for BP management. On the 14th day her BP began to variate between 240/120 and 60/40. She suffered from severe headache and body temperature reached 39.5°C, suggesting signs of PMC. Without using phentolamine, she was directly treated with chlorpromazine (25 mg i.m. followed by 3–4 mg/h continuous infusion, Figure 2D). Glucose saline was also infused for volume expansion. Subsequently, BP fluctuation abated and gradually stabilized between 100/60 and 120/80 mmHg in the next 3 days (Figure 2D). The operation was successful with no complications, and the BP stabilized around 110–120/60–70 mmHg. Her plasma catecholamine metabolite levels returned to normal levels following treatment (metanephrine: <0.07 nmol/L; normetanephrine: 0.22 nmol/L, Supplementary Table 1). Pathological analysis confirmed that the tumor was a PHEO (Figure 1D). Postoperatively, the patient's condition was stable and she was discharged 6 days after surgery.

## Discussion

PMC is a critical and lethal condition in patients with PHEOs. Despite rescue measurements including ECMO renal replacement therapy, the mortality rate remains high (26%) (6). This case report of patients with PMC showed that chlorpromazine application efficiently controlled PMC in all patients, thus providing a potentially effective alternative therapy for BP management in patients with PMC or PHEOs. Three of all four cases (Patient 1–3) we presented exhibited poor responses to phentolamine recommended in classic textbooks during PMC episodes, while chlorpromazine administration resulted in a dramatic control of oscillatory BP during PMC episodes and remained stable with the frequency of hypertensive episodes in all four patients. Dosage of chlorpromazine were determined based on fierceness of patients' BP fluctuation. Speed of chlorpromazine infusion were regulated dynamically as the patients' BP variates. A relatively less severe hypertension were treated with an initial dose of 25 mg intramuscularly followed by slower continuous infusion and higher doses were used if the patients' circumstances were more severe. Moreover, the BP amplitude gradually decreased without irreversible hypotension and was completely stabilized within 3–5 days after chlorpromazine application, with no further PMC episodes. Several days later, all 4 patients become stable, their functions of injured multi-organs recovered to normal and all the patients underwent successful tumor resections. Postoperatively, all 4 patients from our hospital survived, with stabilized BP and normal catecholamine metabolite levels. Additionally, three sporadic PMC cases from the literature also showed similar efficacy of chlorpromazine (16, 17). In 1977, Erkki et al. reported two cases of 14-year-old children with PHEO. The first was a 14-year-old boy with sustained hypertension. Systolic pressure was over 200 mmHg (Supplementary Table 2). Aortography revealed a vascular mass in the left adrenal gland. Treatment of chlorpromazine for a week (25 mg × 4) were used and BP fell to 130–150/70–80 mmHg. Postoperatively, abnormal BP immediately returned to normal (120–130/70–80 mmHg). The second was a 14-year old girl with reddish blue color on the fingers and toes, nocturnal sweating and paroxysmal headache. On admission she had severe hypertension (250/130 mmHg) and tachycardia (110 bpm). Retrograde aortography showed large shadow in the right adrenal gland region and a smaller one in the left adrenal. Patient's BP reduced to 160–170/90–120 mmHg after pretreatment with chlorpromazine (25 mg × 4) for a week (Supplementary Table 2). Soon afterwards, the patient successfully underwent surgical therapy. BP fell to 100–120/70–80 after bilateral adrenalectomy (16). In 1962, Lund-Johansen reported a patient with PHEO. On admission he was cold, sweating with a rectal temperature of 40.2°C. BP was 220/120 mmHg and pulse was 120 bpm (Supplementary Table 2). Urine epinephrine was 1,900 ug/24 h and norepinephrine was 1,680 ug/24. He suffered from identical symptoms 10 years ago but recovered with antibiotics and fluids. He was given 50 mg chlorpromazine intramuscularly followed by 12.5 mg chlorpromazine intramuscular injection every 6 h. His systolic BP stabilized between 120 and 140 mmHg. He also had acute pancreatitis and his doctor decided that his condition was not fit for surgical treatment at that time. However, his condition got worse and he developed pronounced meteorism and permanent urinary retention. Unfortunately, he due to cachectic condition with uremia and anuria (17). Despite the unhappy ending for this patient, this case still support the BP regulating effect of chlorpromazine in PMC episodes. Overall, the seven cases showed that treatment with chlorpromazine reduced the intensity of the hypertensive crisis in patients with PMC, as well as fluctuations and frequencies of hypertensive-hypotensive attacks. Average reduction of maximum systolic BP in the seven patients after chlorpromazine application was 112±49.15 mmHg. These findings suggest that chlorpromazine may be a potentially ideal choice for hemodynamic regulation and PMC prevention or rescue. Thus, further study on the use of chlorpromazine is warranted.

**Figure 3:**
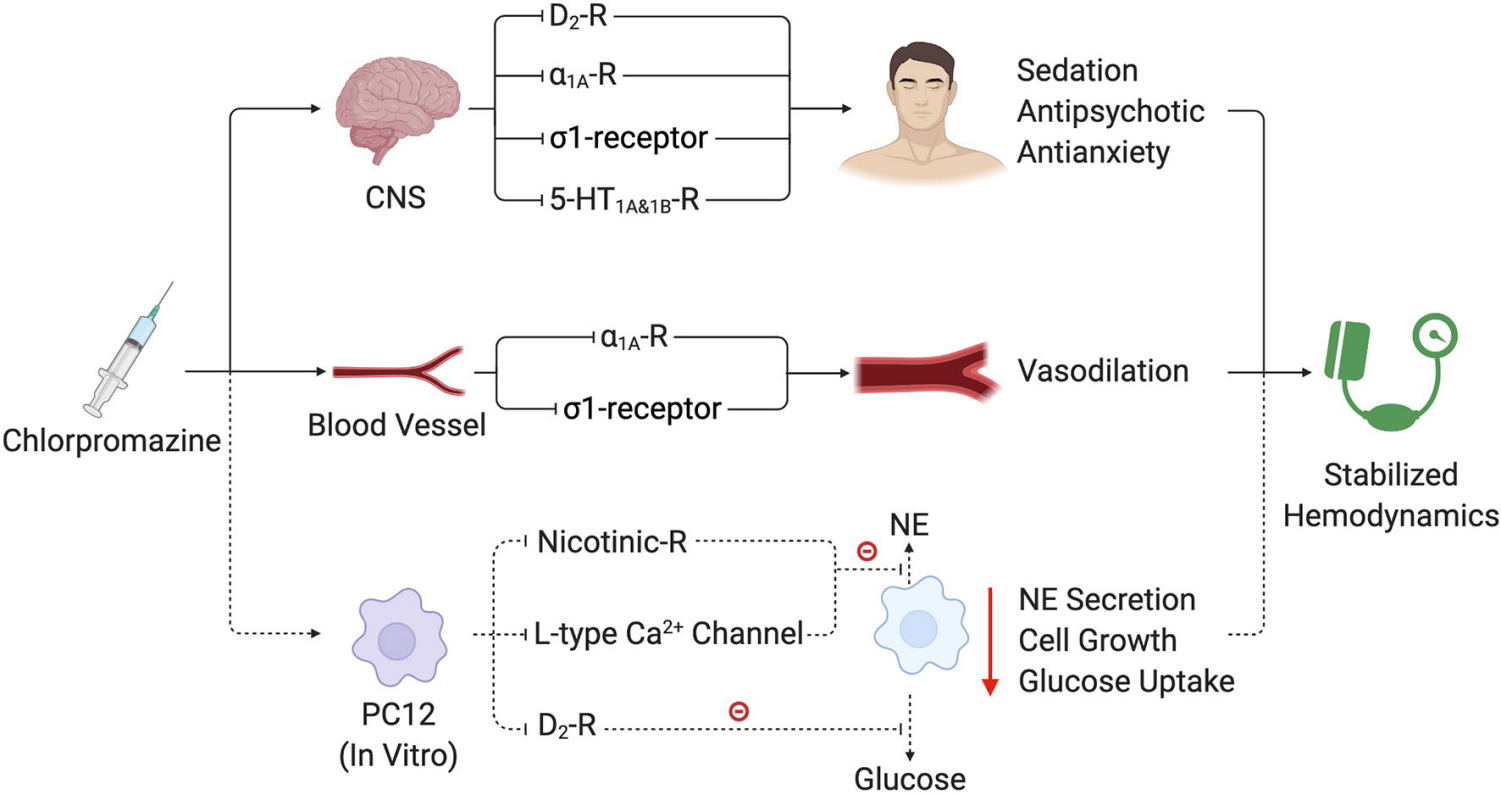
Potential mechanisms of chlorpromazine treating PMC. By blocking various receptors in CNS, blood vessels and tumor cells, chlorpromazine manage to control PMC. Mechanisms that were proven in human are connected with solid lines, while those only tested *in vitro* are connect with dash lines. CNS, central nerve system; PC12, rat pheochromocytoma cells; NE, norepinephrine; R, Receptor.

The potential mechanisms of the therapeutic effect of chlorpromazine are as follows (Figure 3). Central nerve system responses of chlorpromazine including sedation, antipsychotics and antianxiety relieve patients' tension and anxiety. Chlorpromazine exhibits a high affinity for dopamine receptors (mainly dopamine_2_-receptor, D_2_-R), which are primarily responsible for the antipsychotic effect that reduces patient tension and anxiety (20). As an adrenal receptor antagonist, chlorpromazine shows a high affinity to α_1A_- and α_1B_-receptors. Blockade of α_1A_-receptors in the frontal cortex and thalamus also contributes to the antipsychotic effect (Figure 3) (20–22). Inhibition of the 5-hydroxytryptamine receptor (5-HT), specifically 5-HT_1A_ and 5-HT_1B_, plays a role in its anxiolytic activity (15). Chlorpromazine reportedly interacts with σ1-receptors (23). This interaction with σ1-receptors in the medulla may contribute to the sedative effect (24). Decreased systolic BP has been reported in σ1-receptor agonist therapy, suggesting another potential mechanism by which chlorpromazine modifies BP (25). Most importantly, PHEO cells secrete excess catecholamine, predominantly norepinephrine, and activate the α_1_-receptor, causing vasoconstriction that leads to serious hypertension (26). Chlorpromazine antagonizes the overactivated α1-receptor and leads to stable BP reduction during hypertensive episodes (Figure 3) (15). Our observations and previous reports also indicate that the use of chlorpromazine does not result in marked hypotension and it can be easily reversed with regular volume expansion (glucose saline) without the need for extracorporeal membrane oxygenation (ECMO), unlike sodium nitroprusside. In 1980, Robert et al. reported 9 patients (age: 43–61 years), with severe hypertension (>210/130 mmHg) (27). Intramuscular injection of 50 mg chlorpromazine and intravenous infusion of 50 mg furosemide resulted in a gradual and adequate reduction of BP. Mean arterial BP fell by an average of 79 mmHg (42% of initial value) with no side effect (27). Moreover, chlorpromazine reportedly directly affected PHEO cells (Figure 3) (21, 28, 29). Lee et al. demonstrated that chlorpromazine inhibited catecholamine secretion in rat pheochromocytoma (PC12) cells by blocking nicotinic receptors and L-type voltage-sensitive calcium channels (28), while Donard et al. reported that chlorpromazine inhibited glucose uptake and growth of PC12 cells (21, 29). Thus, this direct effect on PHEO may contribute to its hypotensive effect in patients with PHEO.

This study has some limitations. As a case report and literature review, different sources of bias are possible. We searched for similar studies to solidify the evidence of potential beneficial effects associated with chlorpromazine. Since very limited studies reported chlorpromazine usage on PMC, dosage of chlorpromazine were determined empirically and rather cautious. Despite chlorpromazine application, Complete BP stabilization required 3–5 days. An earlier intramuscular injection of 50 mg chlorpromazine and a larger dose (10 mg/h) of continuous infusion may control BP more efficiently. However, further evidence is needed to find out the ideal dose of chlorpromazine for PMC patients.

## Conclusion

Our cases combined with reported sporadic case reports from the literature demonstrated the potential benefit of chlorpromazine for hemodynamic control during PMC episodes, in which all patients BP fluctuation were less severe after chlorpromazine application and 6 (4 from our hospital and 2 from literature report) were able to successfully undergo surgical operations, with good postoperative clinical response. Therefore, we believe that chlorpromazine showed potential for BP control in PMC. Our results suggest a potentially more effective alternative medication to the currently recommended therapy and support further studies on the use of chlorpromazine.

## Data Availability Statement

The raw data supporting the conclusions of this article will be made available by the authors, without undue reservation.

## Ethics Statement

The studies involving human participants were reviewed and approved by Ethics committee of Tongji hospital, Tongji Medical College, Huazhong University of Science and Technology. The patients/participants provided their written informed consent to participate in this study. Written informed consent was obtained from the individual(s) for the publication of any potentially identifiable images or data included in this article.

## Author Contributions

JJW performed patients' data collection, analyzation and interpretation, and wrote the manuscript draft. ZH participated in patients' treatment, data collection, and revised the manuscript. BY revised the manuscript. YY, HW, HD, GC, and LW participated in patients' treatment, rescue and clinical follow-up after surgery. JJ participated in patients' clinical follow-up and revised the manuscript. DW proposed the alternative treatment of chlorpromazine, finalized the manuscript, and correspond to the study. All authors contributed to the article and approved the submitted version.

## Funding

This work was supported in part by key grants from the National Key R&D Program of China (No. 2017YFC0909400) and the National Nature Science Foundation of China (Nos. 81790624 and 81700348).

## Conflict of Interest

The authors declare that the research was conducted in the absence of any commercial or financial relationships that could be construed as a potential conflict of interest.

## Publisher's Note

All claims expressed in this article are solely those of the authors and do not necessarily represent those of their affiliated organizations, or those of the publisher, the editors and the reviewers. Any product that may be evaluated in this article, or claim that may be made by its manufacturer, is not guaranteed or endorsed by the publisher.
