# Supplementary material for: Chlorpromazine Efficiently Treats the Crisis of Pheochromocytoma: Four Case Reports and Literature Review
Source: Front Cardiovasc Med. 2021 Nov 22;8:762371. doi: 10.3389/fcvm.2021.762371 (PMC8645834; doi:10.3389/fcvm.2021.762371)
Supplement: Supplementary file 1 [file Table_1.DOCX]

**Supplementary Table S1. Patients’ clinical characteristics**

| Clinical Index | Patient 1 | Patient 2 | Patient 3 | Patient 4 | Reference Value |
| --- | --- | --- | --- | --- | --- |
| Age | 42 | 57 | 40 | 57 | - |
| Gender | Male | Female | Female | Female | - |
| Blood Pressure (mmHg) | 100/60~330/150 | 80/50~270/130 | 80/40~179/111 | 60/40~240/120 | - |
| Blood Pressure* (mmHg) | 98/61~126/80 | 100/55~116/60 | 100/50~110/60 | 110/60~120/70 | - |
| Pulse (bmp) | 100 | 121 | 150 | 110 | 60~100 |
| Pulse* (bmp) | 61~88 | 69~80 | 70-80 | 79~85 | 60~100 |
| **Urine Tests** |  |  |  |  |  |
| Norepinephrine (ug/24h) | 1927.2 | - | 300.3 | 212.8 | 1~100 |
| Norepinephrine* (ug/24h) | - | 18.3 | 15.3 | - | 1~100 |
| Epinephrine (ug/24h) | 73 | - | 793 | 334 | 0~14 |
| Epinephrine* (ug/24h) | - | 5.5 | 2.3 | - | 0~14 |
| VMA (umol/24h) | 453.6 | 409.2 | 110.2 | 65.2 | 0.0~41.28 |
| VMA* (umol/24h) | - | 25.2 | 14.8 | - | 0.0~41.28 |
| **Imageological Examination** |  |  |  |  |  |
| Side | Left | Right | Left | Left |  |
| Ultrasonography imaging (cm) | 8.7 x 8.5 | 5.1 x 3.4 | 3.5 x 3.1 | - |  |
| Computed tomography (cm) | 8.9 x 8.1 | 4.9 x 2.9 | 2.7 x 3.6 | 2.6 x 2.8 |  |
| **Blood Tests** |  |  |  |  |  |
| Normetanephrine (nmol/L) | 41.4 | - | 6.01 | 1.45 | 0~0.59 |
| Normetanephrine* (nmol/L) | 0.08 | - | 0.13 | 0.22 | 0~0.59 |
| Metanephrine (nmol/L) | 1.36 | - | 18.6 | 2 | 0~0.21 |
| Metanephrine* (nmol/L) | <0.07 | - | 0.11 | <0.07 | 0~0.21 |
| WBC (x10^9/L) | 9.42 | 11.44 | 23.79 | 13.23 | 3.50~9.50 |
| Neutrophil (%) | 94.7 | 83.5 | 93.7 | 86 | 40.00~75.00 |
| Lymphocyte (%) | 3.9 | 9.8 | 3.7 | 7.6 | 20.0~50.0 |
| RBC (x10^12/L) | 4.95 | 5.6 | 5.63 | 5.76 | 3.80~5.10 |
| Hb (g/L) | 140 | 165 | 162 | 164 | 115.0~150.0 |
| Plt (x10^9/L) | 154 | 266 | 293 | 157 | 125~350 |
| Fib (g/L) | 5.77 | 5.15 | 2.39 | 5.77 | 2.00~4.00 |
| D-dimer (ug/mL) | 1.78 | 3.24 | 1.68 | 1.78 | 0~0.5 |
| CRP (mg/L) | 7.8 | 36.5 | 8.4 | - | 0.1~3.0 |
| PCT (ng/mL) | - | 0.62 | - | 0.17 | 0.02~0.05 |
| NT-proBNP (pg/mL) | 778 | 34082 | 11596 | 31018 | 0~247 |
| High-sensitivity troponin I (pg/mL) | 371.3 | 153.1 | 27429.5 | 212 | 0~15.6 |
| CK (U/L) | 94 | - | 279 | - | 3~170 |
| Cr (umol/L) | 122 | 77 | 138 | 73 | 45~84 |
| BUN (mmol/L) | 8.46 | 14.06 | 7.38 | 11.3 | 2.6~7.5 |
| UA (umol/L) | 782.9 | 549 | 368 | 252.4 | 142.8~339.2 |
| HCO_3_^-^ (mmol/L) | 25.3 | 18.6 | 18.6 | 28.2 | 22.0-29.0 |
| AST (U/L) | 166 | 38 | 65 | 163 | 0~33 |
| ALT (U/L) | 78 | 34 | 47 | 67 | 0~32 |
| ALB (g/L) | 50 | 48.7 | 41.6 | 34.4 | 35.0~52.0 |
| Alkaline Phosphatase (U/L) | 170 | 96 | 54 | 175 | 6-42 |
| ү-glutamyl transpeptidase | 123 | 27 | 32 | 84 | 10-71 |
| Tbil (umol/L) | 22.7 | 14.6 | 18.5 | 3.9 | 0~21 |
| Dbil (umol/L) | 7.8 | 3.1 | 4.2 | 2.4 | 0~8 |
| Ibil (umol/L) | 14.9 | 11.5 | 14.3 | 1.5 | 0~12.9 |
| LDH (U/L) | 169 | - | 642 | 187 | 134~214 |
| Na (mmol/L) | 141.8 | 138.4 | 138.3 | 144.9 | 135~145 |
| K (mmol/L) | 4.33 | 2.9 | 3.98 | 2.85 | 3.50~5.10 |
| Cl (mmol/L) | 99.3 | 93.7 | 95.4 | 98.5 | 99~110 |
| Ca (mmol/L) | 2.62 | 2.03 | 2.28 | 2.27 | 2.15~2.50 |

Values with * indicate values after surgery. Values without * indicate values before surgery and treatment with chlorpromazine and volume expansion. Abbreviations: VMA, vanillylmandelic acid; WBC, white blood cell; RBC, red blood cell; Hb, hemoglobin; Plt, platelet count; Fib, fibrinogen; CRP, C-reactive protein; PCT, procalcitonin; NT-proBNP, amino-terminal pro-brain natriuretic peptide; CK, creatine kinase; Cr, creatinine; BUN, blood urea nitrogen; UA, uric acid; AST, aspartate aminotransferase; ALT, alanine aminotransferase; ALB, albumin; TBil, total bilirubin; Dbil, direct bilirubin; Ibil, indirect bilirubin; LDH, lactic dehydrogenase.

**Supplementary Table S2. Reviewed patients’ BP variation**

|  | **Reviewed Patient 1** | **Reviewed Patient 2** | **Reviewed Patient 3** |
| --- | --- | --- | --- |
| BP (mmHg) | 200/110~230/120 | 210/150~230/160 | 215/100~220/120 |
| BP* (mmHg) | 120/70~150/80 | 120/40~130/80 | 120/100~140/- |
| BP** (mmHg) | 110/160~140/70 | 100/70~140/90 | - |

BP indicate blood pressure before application of chlorpromazine. BP with * indicate blood pressure 3-5 days after chlorpromazine application. BP with ** indicate blood pressure after tumor extirpation.
